# Cholangiocarcinoma Presenting with Hypercalcemia and Thrombocytopenia

**DOI:** 10.1155/2014/246817

**Published:** 2014-06-15

**Authors:** Muharrem Battal, Bünyamin Gürbulak, Ozgür Bostanci, Müveddet Banu Yılmaz, Yasar Ozdenkaya, Oguzhan Karatepe

**Affiliations:** ^1^General Surgery Department, Sisli Etfal Teaching and Research Hospital, Sisli, 34173 Istanbul, Turkey; ^2^Arnavutkoy Government Hospital General Surgery Department, Arnavutkoy, 34555 Istanbul, Turkey; ^3^Pathology Department, Sisli Etfal Teaching and Research Hospital, Sisli, 34173 Istanbul, Turkey; ^4^Medipol University Hospital General Surgery Department, Bagcılar, 34214 Istanbul, Turkey; ^5^Bezmi Alem University Hospital General Surgery Department, Fatih, 34093 Istanbul, Turkey

## Abstract

Malignant hypercalcemia and thrombocytopenia may result from bone metastasis of cholangiocarcinoma (CC). Our case was 53-year-old man admitted to emergency department with symptoms of anorexia, weight loss, nausea, vomiting, and general fatigue in February 2012. His laboratory findings showed hypercalcemia and thrombocytopenia. CT showed a large multinodular mass in the right lobe and, extending through left lobe of the liver. We considered the diagnosis of hypercalcemia of malignancy with elevated calcium levels and suppressed PTH level with the existence of skeletal bone metastasis and the absence of parathyroid gland pathology. Treatment of hypercalcemia with IV saline, furosemide, and calcitonin improved the patient symptoms. After the 8th day of admission, calcium level, thrombocytopenia, and other symptoms were normalized. Patient was sustained surgically inoperable and transferred to medical oncology department for the purpose of palliative chemotherapy and intended radiotherapy for bone metastasis. Hypercalcemia relapsed 4 weeks after discharge and patient died at the 5th month after admission due to disseminated metastasis. We should be aware of CC with symptomatic hypercalcemia and rarely low platelet count. The correction of hypercalcemia provides symptomatic relief and stability of patients.

## 1. Introduction

CC is a well-known malignancy which has a poor prognosis. Its prevalence has been estimated to be 5.3% in patients with hepatocellular carcinoma (HCC) and 17.5% in patient with CC [1]. Generally, hypercalcemia with an advanced disease and the interval between the discovery of hypercalcemia and the patient death is typically less than one year [2]. Although chemotherapy for CC is administered to inoperable patients, the results are largely disappointing [3].

## 2. Case Presentation 

**Figure 1:**
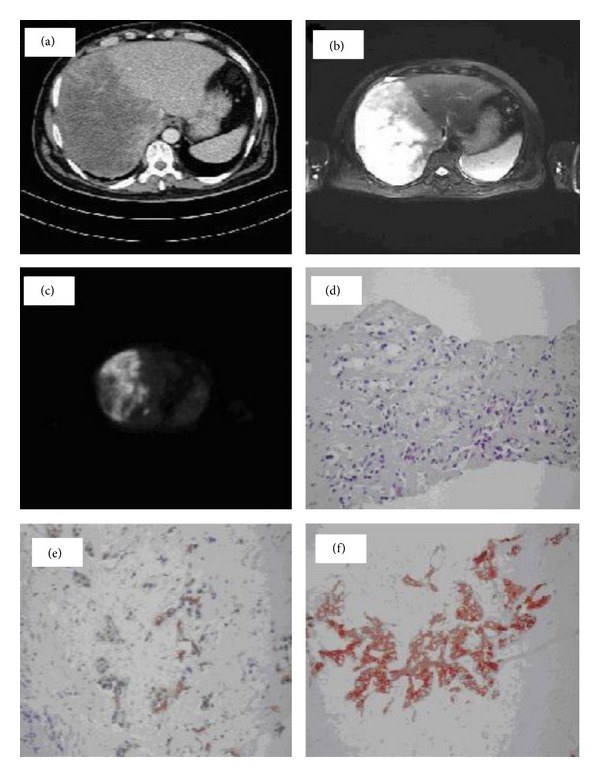
(a) CT scan, 20 × 14 cm size hypoattenuating multinodular tumor mainly filling the right lobe of the liver with irregular boundaries. (b) MR, T2-weighted images as a slightly hyperintense area with contrast enhancement of peripheral zone being liable to confluent multiple lesions in the right lobe of liver. (c) Fluorodeoxyglucose-positron emission tomography (FDG-PET) showed 10.7 of a standardized uptake value for FDG in CC of liver. (d) Tumor cells diffusely formed ductal structures in fibrotic stroma and have a big hyperchromatic nucleus in narrow cytoplasm, H&E, ×200. (e) Tumor cells diffusely replaced the liver parenchyma and formed ductal structures in fibrotic stroma which were positively stained for cytokeratin 19 immunoreaction, ×200. (f) Tumor cells diffusely replaced the liver parenchyma and formed ductal structures in fibrotic stroma which were positively stained for cytokeratin 7 immunoreaction ×200.

53-year-old man was admitted to our clinic at February 2012 with symptoms of general fatigue, anorexia, weight loss, nausea, and vomiting. His past history was unremarkable and he only complained of 20 kilos' weight loss extending over 4 months. Physical examination was unremarkable other than an enlarged liver extending to 5 cm below the right costal margin. Laboratory tests showed WBC of 9430 K/*μ*L, hbg of 12.4 g/dL, hct of 36.2%, plt of 2000 K/*μ*L, calcium of 14.9 mg/dL (8.6–10.2), phosphate of 1.8 mg/dL (2.6–4.5), albumin of 3.4 g/dL, alkaline phosphatase (ALP) of 295 (40–130), and gamma-glutamyl transferase (GGT) of 363 U/L (0–60). PTH was suppressed to 5 pg/dL (15–65), and CA 19-9 was as high as 2384 U/mL (<27). Patient consulted endocrinology and hematology departments and medical treatment was applied. Peripheral blood smear staining showed thrombocytopenia. Thorax and abdominal CT, abdominal MRI, and FDG-PET studies were performed. Abdominal USG showed a large tumor with hypoechogen pattern in right lobe of the liver. CT revealed 20 × 14 cm size hypoattenuating multinodular tumor mainly filling the right lobe of the liver with irregular boundaries (Figure 1(a)). Thorax and brain CT were normal. T1-weighted MR images showed the tumor as a hypointense area and T2-weighted images showed it as a slightly hyperintense area with contrast enhancement of peripheral zone being liable to confluent multiple lesions in right lobe of liver with retrocaval and aortocaval multiple metastatic lymphadenopathy and metastatic lesions on right iliac and right femoral bones (Figure 1(b)). Fluorodeoxyglucose-positron emission tomography (FDG-PET) showed pathological signal intensity of 10.7 of a standardized uptake value in CC of liver, in renal hilum, in bilateral aortocaval lymph nodes, and in six places of bones as C-5, T-11, and S-1 vertebral corpus, right iliac bone, and left and right ischium (Figure 1(c)).

**Figure 2:**
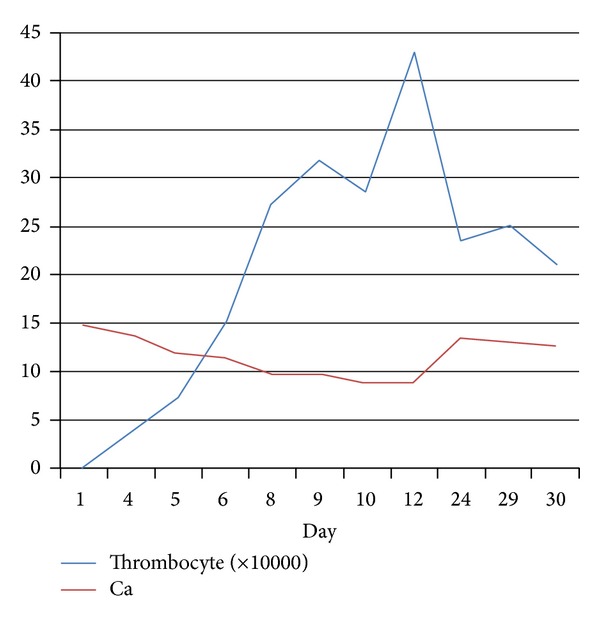
After treatment, relationship with calcium and thrombocyte level.

The patient was treated with IV saline, furosemide, and calcitonin. After the 8th day of treatment, calcium level, thrombocytopenia, and other clinical symptoms were normalized (Figure 2). After the patient stability and normal platelet count were obtained, we performed USG guided trucut liver biopsy. USG guided trucut liver biopsy confirmed the diagnosis of CC. Neoplastic cells stained positively by cytokeratins 7 and 19. Patient was evaluated surgically as inoperable with evidence of radiological and pathological findings and was directed to medical oncology department for purpose of palliative chemotherapy with intended radiotherapy for bone metastasis (Figures 1(d), 1(e), and 1(f)).

At the end of first month of the oncologic treatment, patient was readmitted to hospital with worsening of consciousness state and relapsed hypercalcemia (11.5 mg/dL), while thrombocyte count was normal. Calcium level was higher than normal levels but not as high as first admittance to lead to thrombocytopenia again. Medical treatment of hypercalcemia rapidly stabilized the patient consciousness state. In spite of the chemoradiation treatment, disseminated metastasis developed and patient died in the 5th month of diagnosis.

## 3. Discussion

CC is a rare neoplasm which accounts for approximately 3% of the gastrointestinal cancers worldwide [4]. Although surgical resection remains the only curative treatment, most patients with CC are not operable candidates [3].

Hypercalcemia is a serious and frequent complication and a sign of advanced stage of malignant disease, occurring in 10% to 20% of patients with malignancies [5]. It has been estimated to be of 5.3% in patients with HCC and 17.5% in patients with CC [6]. Approximately 85% of patients with cancer and hypercalcemia have metastatic bone disease. The remaining 15% have some other etiology for hypercalcemia as paraneoplastic syndrome [7]. Humoral hypercalcaemia of malignancy (HHM) is characterised by elevated calcium, low serum phosphorus, low PTH, and low vitamin D levels [8]. The type of hypercalcemia has been associated with the abnormal secretion of various proteins, including PTH-rP by the tumor cells [7, 8]. HHM is usually manifested as certain type of malignancies and is generally associated with squamous-cell carcinomas of esophagus, lung, head, and neck. HHM is rarely seen with CC [9]. The present case had multiple metastatic bone lesions. So, predominantly, these lesions were thought of as the reason for hypercalcemia. And the mechanism of hypercalcemia had no effect on treatment of our case, so we did not need to differentiate etiology of hypercalcemia.

Medical treatment of hypercalcemia is composed of IV hydration with saline, loop diuretics, and antiresorptive agents such as calcitonin and bisphosphonates [10, 11].

Thrombocytopenia in these cases may occur for many reasons and can be seen in some liver tumors like angiosarcomas and hemangiomas. The relationship between hypercalcemia and thrombocytopenia is not clear but in literature it is stated that there is correlation between hypercalcemia and hypophosphatemia. Hypophosphatemia also impairs granulocyte function by interfering with ATP synthesis, increasing platelet diameter, and shortening platelet survival so it triggers the marked platelet disappearence. And these lead to thrombocytopenia and reactive megakaryocytosis [12]. In this case thrombocyte level was as low as 2000 K/*μ*L. But there was not any spontaneous bleeding. So we did not need transfusion. The patient was treated with IV saline, diuretics, and calcitonin.

## 4. Conclusion 

We presented a case of metastatic CC with symptomatic hypercalcemia, hypophosphatemia, and low platelet count. With increased calcium levels and suppressed PTH with existence of metastatic bone disease clinician should recognize this rare presentation of CC. The correction of hypercalcemia and hypophosphatemia provides correction of thrombocytopenia.
